# Case report: Unusual persistent hypotension and acute occlusion after peripheral paclitaxel balloon angioplasty

**DOI:** 10.3389/fcvm.2022.964601

**Published:** 2022-10-14

**Authors:** Lin Yang, Jianlin Liu, Chao Liu

**Affiliations:** Department of Vascular Surgery, First Affiliated Hospital of Xi'an Jiaotong University, Xi'an, China

**Keywords:** paclitaxel-coated balloon, no-reflow phenomenon, femoropopliteal artery occlusion, persistent hypotension, complication

## Abstract

**Background:**

Paclitaxel-coated balloon (PCB) angioplasty is a mainstream treatment for peripheral artery disease; however, the safety of PCB remains controversial.

**Case presentation:**

We confirmed acute occlusion during PCB angioplasty in a patient with femoropopliteal artery occlusion. The occluded vessels were revascularized completely after endovascular medical therapy and bailout stenting angioplasty. Then, the patient experienced persistent post-procedure orthostatic hypotension (30 days) and received hydration and vasoactive agents with a target mean arterial blood pressure of 75–85 mmHg. The patient's blood pressure gradually recovered over the 30 days after the procedure, and there was no recurrence of symptomatic hypotension during the follow-up.

**Conclusions:**

This rare complication is helpful to evaluate the safety of the PCB device.

## Introduction

Percutaneous balloon angioplasty is an effective strategy for femoropopliteal artery occlusion; however, restenosis occurs in 40–60% of patients within 1 year, leading to therapeutic failure and reintervention (1–4). Paclitaxel-coated balloon angioplasty (PCB) is a relatively newer endovascular therapy strategy that has been designed to limit the intimal proliferation of target lesions and has been proven to be effective for the treatment of peripheral artery disease (5–7). However, a recent report showed an increased long-term risk of death following the application of paclitaxel-coated balloon angioplasty (8). Paclitaxel is a cytotoxic drug that inhibits smooth muscle proliferation and has potential adverse effects, such as hypotension and arrhythmia (9). Moreover, PCB angioplasty might cause a no-reflow phenomenon or acute occlusion in the target vessel, which is often reported in percutaneous coronary interventions (10, 11); however, there is still no literature reporting persistent hypotension after paclitaxel-coated balloon angioplasty for peripheral artery disease.

## Case presentation

A 63-year-old man with femoropopliteal artery occlusion was admitted to the hospital with intermittent right lower limb claudication for 13 years and exacerbation of ulcers for 5 months. Local ultrasound indicated that the middle of the superficial femoral artery was occluded; the popliteal artery and artery below the knee had severe stenosis. The patient had no history of hypertension, diabetes or cardiovascular disease. He had suffered an ischemic stroke 2 years ago and had experienced mild weakness in the left lower limb; the other risk factor identified was that the patient had smoked for 30 years (20–40 cigarettes/day). Physical examination revealed that the ulcers in the foot and ankle with exudation were categorized as Rutherford V, and the clinical classification of the patient was Fontaine 4. The patient was intolerant of ischemic pain and experienced difficulty lying supine (he was administered flurbiprofen and pethidine); thus, he refused computed tomography angiography. Drug gene sensitivity tests before the procedure suggested that the aspirin genes GPIIIaP1A2 (T > C), PTGS1 (−842A > G), and PEAR1 (G > A) were homozygous wild type, and the clopidogrel gene PON1 (G > A) was homozygous mutant CYP2C19 ^*^ 2 (G > A) and homozygous wild-type CYP2C19 ^*^ 3 (G > A), suggesting that the patient was sensitive to aspirin and had clopidogrel resistance. The statin gene 67SLCO1B1 ^*^ 5 (T > C) was homozygous wild type, suggesting that statins could be used normally. After the necessary preparations and drug therapy (aspirin 100 mg/day, cilostazol 200 mg/day, and statin 20 mg/day), the patient underwent angiography and angioplasty under general anesthesia. Heparin was used during the procedure at an initial dose of 100 U/kg, followed by additional doses as necessary.

**Figure 1:**
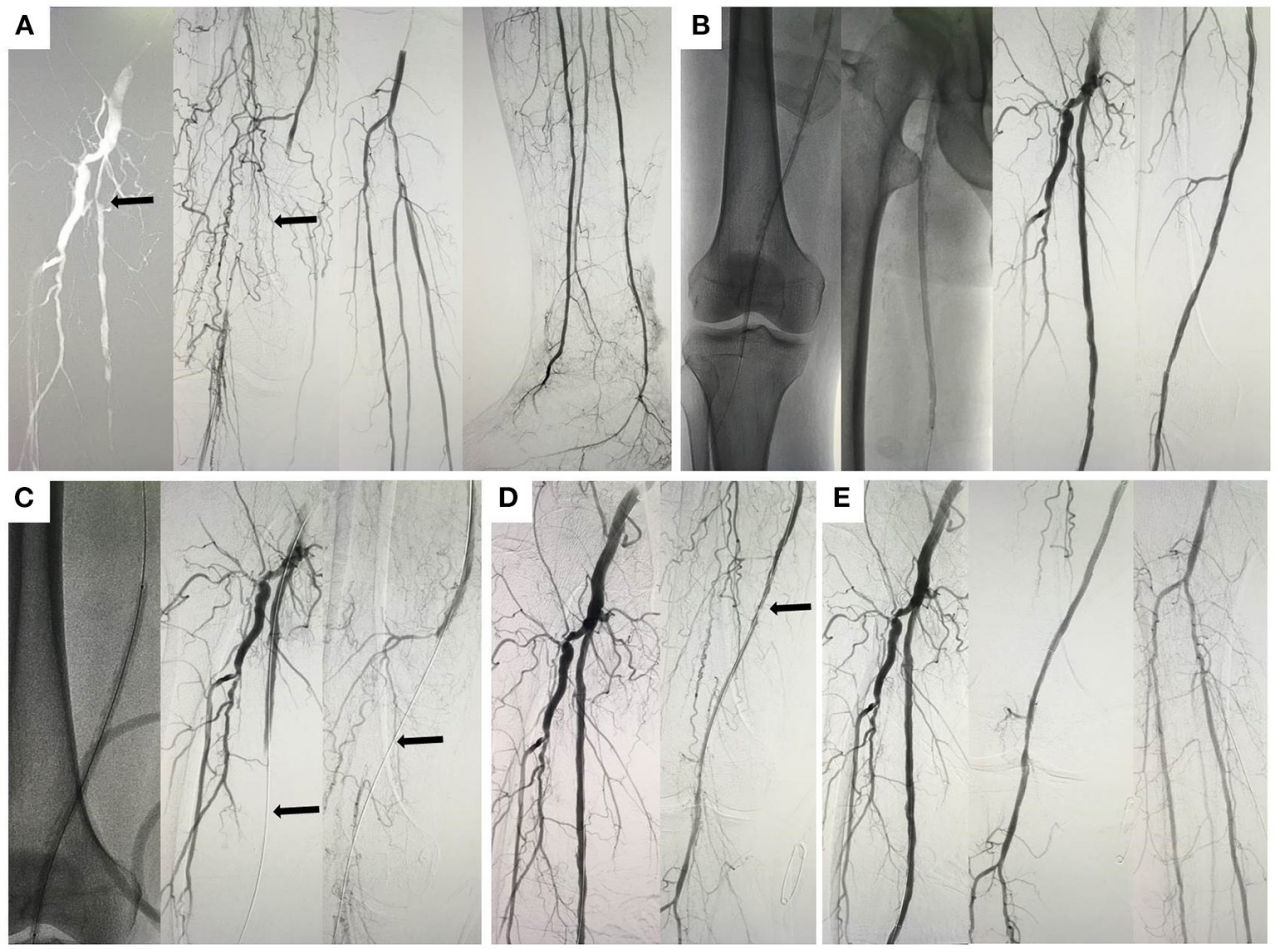
Angiographic images of the patient. **(A)** Initial angiography of the target lesion (black arrow); **(B)** predilation of the lesion and post-dilation images; **(C)** paclitaxel-coated balloon and the no-reflow phenomenon (black arrow); **(D)** angiographic images after intravascular therapy (black arrow); **(E)** final images and blood flow revascularization.

**Figure 2:**
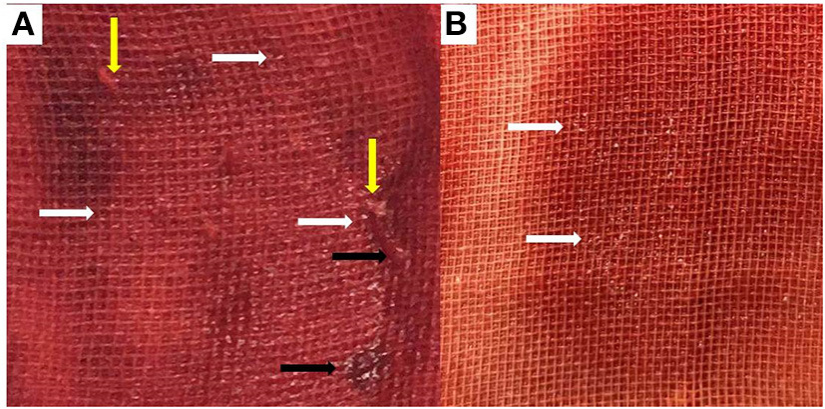
The mixed components of drug debris, thrombosis, and plaque. The mixed components include drug debris [**(A,B)**, white arrow], plaque debris [**(A)**, yellow arrow], and thrombosis debris (black arrow).

Initial angiography showed that the long occlusion of the femoropopliteal artery (~45 cm) and the outflow below the knee were acceptable (Figure 1A). With the support of a long sheath, a 4F catheter and a V-18 (Boston Scientific, Natick, Mass) wire were passed through the occlusion lesion into the distal true lumen. Then, predilation was performed with a plain balloon (Cordis Savvy Long 2, 3, and 4–220 mm and ev3 Evercross 5–200 mm), and angiography indicated revascularization of target vessels without obvious dissection (Figure 1B). Finally, the target vessels were dilated with a PCB (Acotec 5–200 and 5.5–300 mm, 8 atm, 90 s), and acute occlusion occurred after deflation (Figure 1C). Since this was the first case in western China, the reported experience of treating acute occlusion in coronary lesions was used as a basis for treatment. Prior to treatment, we vacuumed the blood from the sheath and found mixed paste-like components of drug debris, plaque and thrombosis (Figure 2), and then rapid injection of urokinase (300,000 units), papaverine (60 mg), and heparin saline (250 ml) through the arterial catheters was performed; this strategy was repeated after 15 min. Thereafter, angiography confirmed that the lesion was reopening, but a flow-limiting dissection was detected distal to the femoral artery; thus, a bailout stent (Cordis Smart Control, 6–100 mm) was placed (Figure 1D). Final angiography showed that the dissection had disappeared and that the blood flow had returned to normal (Figure 1E). The patient was transferred to the intensive care unit.

**Figure 3:**
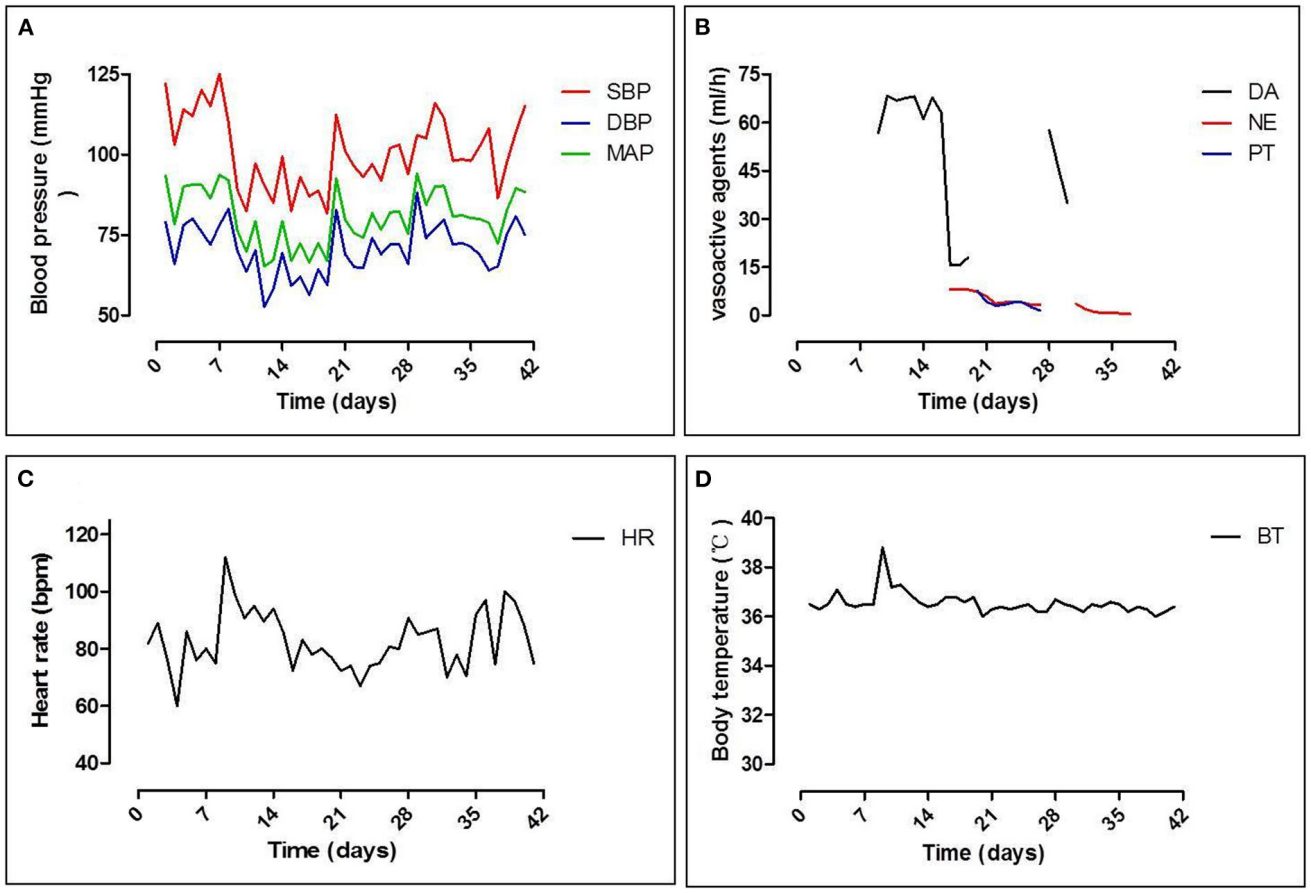
Dynamic changes in vital signs. **(A)** Changes in systolic pressure (SBP), diastolic pressure (DBP), and mean arterial pressure (MAP); **(B)** vasoactive agents used during hypotension: dopamine (DA), norepinephrine (NE), and posterior pituitary hormone (PT); **(C)** changes in heart rate; and **(D)** body temperature.

The patient's systolic blood pressure (SBP) fluctuated between 80 and 95 mmHg following the procedure (Figure 3A). Dopamine (DA, 40–75 ml/l) was administered to maintain the patient's blood pressure in the first week, and then the DA concentration was gradually reduced (Figure 3B). However, the patient suffered sudden orthostatic hypotension (DA, 30 ml/l) when arising from bed and was immediately given resuscitation therapy (DA, 70 ml/l). The patient recovered completely after 2 min, and his SBP gradually stabilized at 90 mmHg (Figure 3A). To avoid the side effects of DA, the concentration was reduced at post-procedure day 9, and noradrenaline (NE) was administered (starting concentration: 8 ml/h, Figure 3B). NE (8 ml/h) and posterior pituitary hormone (PT, 10 ml/h) were administered on day 12. When the patient's SBP stabilized, the concentrations of NE and PT were gradually reduced to 3 and 1.5 ml/h, respectively (day 20). Owing to the vasoconstrictive effect of PT, the skin at the infusion site darkened, and then the PT and NE were replaced with DA (concentration, 30 ml/h−100 ml/l); however, the patient's SBP was still difficult to control, and NE was reintroduced after 3 days (day 23). The patient's SBP gradually stabilized between 90 and 100 mmHg, and all vasoactive drugs were stopped on day 30. Interestingly, the heart rate and body temperature of the patient remained stable during the entire therapeutic process (Figures 3C,D).

Although the blood culture (blood samples and ulcer secretions) was negative, biochemical results confirmed the inflammatory status indicated by ulcer exudation. After the initiation of treatment, the levels of the indicators decreased and stabilized (Table 1). The patient developed transient elevation of myocardial enzyme levels, although the patient had no symptoms of cardiovascular disease, and the levels of the indicators improved after therapy. The platelet counts of the patients were normal during the whole process, the antibody of heparin-induced thrombocytopenia was negative (Table 1), and no deep vein thrombosis occurred under ultrasound examination.

**Table 1:** Clinical laboratory results.

**Items**	**Reference range**	**Day 0**	**Day 1**	**Day 7**	**Day 15**	**Day 22**
White-Cell count (10^9^/l)	3.5–9.5	14.78	17.13	10.15	10.36	9.72
Red-Cell count (10^12^/l)	4.3–5.8	4.01	3.44	3.25	3.45	3.43
Absolute neutrophil count (10^9^/l)	1.8–6.30	12.53	15.04	8.58	10.36	6.77
Absolute lymphocyte count (10^9^/l)	1.1–3.2	1.4	1.01	0.94	1.55	1.81
Platelet count (10^9^/l)	125–350	460	350	363	445	320
Hemoglobin (g/l)	130–175	124	104	101	103	101
Sodium (mmol/l)	137–147	134	145	138	144	144
Potassium (mmol/l)	3.5–5.3	4.05	3.69	2.98	3.43	3.08
Chloride (mmol/l)	96–108	89.8	105.1	98.4	106.9	102.6
Calcium (mmol/l)	2.11–2.52	2.02	1.83	1.81	2	1.94
Carbon dioxide (mmol/l)	22–29	17	21.6	22.2	15	20
Anion gap (mmol/l)	–	34.2	24.7	23	28.5	27.3
Glucose (mmol/l)	3.9–6.1	3.41	4.51	6.97	4.81	4.17
Blood urea nitrogen (mmol/l)	3.6–9.5	5.69	2.21	3.64	3.85	3.31
Creatinine (umol/l)	57–111	54	45	46	66	44
Total protein (g/l)	65–85	68.2	47.1	54.4	63.9	59.3
Albumin (g/l)	40–55	36	25.3	27.1	34.7	33.6
Total bilirubin (umol/l)	3.4–17.1	6.8	8.1	7.2	4.2	5.9
Procalcitonin (<0.5 ng/ml)	<0.5	0.093				
Alanine aminotransferase (U/l)	9–50	115	88	105	62	71
Aspartate aminotransferase (U/l)	15–40	51	55	70	33	31
Alkaline phosphatase (U/l)	45–125	286	215	280	206	162
Fibrinogen (g/l)	2–4	6.83	5.4	7.52	8.12	4.43
Lactate dehydrogenase (U/l)	120–250	–	256	229	–	–
Prothrombin time (s)	11–14	12.4	16.8	13.8	13.7	12.4
International normalized ratio	0.94–1.30	0.95	1.38	1.08	1.07	0.94
Creatine kinase (U/l)	50–310	–	454	68	–	–
C-Reactive protein (mg/l)	0–10	168	–	100	70	–
Antibody of heparin-induced thrombocytopenia	(–)	NA	(–)	(–)	(–)	NA

## Discussion

A previous report demonstrated that PCB angioplasty results in a lower rate of target lesion revascularization at 24 months compared with traditional balloon angioplasty (17 vs. 52%) (10). The case confirmed mid-term patency for up to 2 years, and restenosis then occurred beyond the distal primary stent. The most important prerequisite for PCB angioplasty is the preparation of the target vessels. No flow-limiting dissection or elastic retraction was detected post-dilation, and PCB angioplasty was performed. These points were closely related to the outcome of PCB.

This patient suffered from acute occlusion after PCB angioplasty following adequate vessel preparation, and this was the first case in Western China; we treated the patient according to the cardiologists' experience and reconstructed the blood flow. Thereafter, we reviewed the images from the procedure and related literature pertaining to coronary artery disease. The potential reasons for acute occlusion in this patient are as follows: (I) thrombosis: Pathological studies in coronary artery disease have reported that the fibrous cap can rupture, leading to the formation of a thrombus and acute occlusion (11). In this case, the plaque lesion might have ruptured following PCB angioplasty, thus explaining why thrombolytic therapy was effective. Furthermore, inadequate anticoagulation or antiplatelet therapy may lead to acute thrombosis occlusion during the procedure. Therefore, we recommend that sufficient heparinization and appropriate antiplatelet drugs be used according to the results of the drug genetic tests. (II) Plaque debris embolization: The microembolization of debris is an important factor in the pathogenesis of acute occlusion (12, 13). Due to the different features of lesions, especially in mixed plaques, distal embolism may be caused during dilation, leading to acute occlusion and secondary thrombosis, which may occur in the distal vessel bed. (III) Dissection: A previous report indicated that severe dissection was found in 42% of cases after dilation, and flow-limiting dissection could cause acute occlusion and affect the outcome of PCB (14). In this patient, dissection was confirmed and treated with a self-expandable stent after endovascular therapy. (IV) Microvascular ischemia and edema and arterial endothelium were stimulated by paclitaxel or the allergic reaction to paclitaxel/excipient, which may have resulted in prolonged ischemia, endothelium edema and impaired microcirculation. (V) Crushed ice effect: The balloon was coated with paclitaxel and low levels (~1%) of an excipient (magnesium stearate) during the manufacturing process (15). In this case, the level of magnesium stearate was 2%. A higher level would result in improper lubrication and decrease the rate of dissolution of the drug (16). Paclitaxel acts on the vessel wall during inflation, which may produce debris, and debris is dissolved in a process similar to how crushed ice gradually melts in warm water. The mixed components might produce a paste-like consistency, and acute occlusion may occur (Figure 2).

The patient presented with persistent hypotension for 30 days following PCB angioplasty and persistent orthostatic hypotension. The systolic pressure of the patient recovered to 90–100 mmHg after 30 days of intravenous therapy, and the patient was then discharged from the hospital. The patient's blood pressure did not return to the pre-procedure average level within 1 year. Obviously, the patient developed drug-induced autonomic nervous system dysfunction. This rare complication has not yet been reported in patients undergoing PCB angioplasty; it has been reported in only one patient who underwent chemotherapy with paclitaxel and cisplatin (9). Compared with the large dose of paclitaxel (200 ± 250 mg/m^2^) used in chemotherapy patients, the amount of paclitaxel used in PCB angioplasty is very small (3.3 ug/mm^2^) (15); however, the lower dose of paclitaxel could still potentially affect the sympathetic control of blood pressure (17, 18). This severe complication may be a potential danger posed by the application of PCB angioplasty for peripheral artery disease and partially explain the increase in all-cause mortality caused by PCB angioplasty (8). However, the mechanism of this rare complication still requires further investigation, especially the effect or allergic reaction of paclitaxel/excipients on the arterial endothelium system.

Our limited experience with one patient cannot be extrapolated to all patients undergoing PCB angioplasty. However, pre-procedure drug genetic testing, aggressive intravenous hydration and vasoactive agent therapy are essential for recovery from acute occlusion and hypotension in patients treated with PCB angioplasty.

## Data availability statement

The original contributions presented in the study are included in the article/supplementary material, further inquiries can be directed to the corresponding author.

## Ethics statement

The studies involving human participants were reviewed and approved by the First Affiliated Hospital of Xi'an Jiaotong University Review Board. The patients/participants provided their written informed consent to participate in this study and for the publication of this case report.

## Author contributions

LY, JL, and CL: data analysis, interpretation, data collection, writing, and literature search. LY and CL: data analysis, interpretation, study design, and data collection. LY: data collection, data analysis, writing, reviewing, and study design. All authors have read and approved the final manuscript.

## Conflict of interest

The authors declare that the research was conducted in the absence of any commercial or financial relationships that could be construed as a potential conflict of interest.

## Publisher's note

All claims expressed in this article are solely those of the authors and do not necessarily represent those of their affiliated organizations, or those of the publisher, the editors and the reviewers. Any product that may be evaluated in this article, or claim that may be made by its manufacturer, is not guaranteed or endorsed by the publisher.
